# More Letters of a Layman: A Postscript

**Published:** 1921-07-16

**Authors:** H. L. Brunswick


*To the Editor of* The Hospital.

### More Letters of a Layman: A Postscript.

*To the Editor of* The Hospital.

Sir,—The instructive "Letters of a Layman" in your issue of July 9 could stand a general postscript. Just as surely as one expects to find a nursemaid behind the perambulator, so must one also anticipate an effulgence following an Editor. . . . But frequently it is not all the patient's fault if, upon placing himself in the care of the doctors, he feels he is unsafely in the hands of the Philistines. For, as a rule, the medical man is too diffident in presenting his point of view. He will generalise instead of definitely stating an opinion which would soothe the troubled mind of his patient.

A doctor should seek to know and understand his patients, otherwise should the latter be a victim of chronic lassitude he would have no small amount of difficulty in diagnosing a mere depression which will pass. Again, it is not untoward to imagine that a man who suffers from abdominal pains might attach the same importance to interiors as his doctor, provided the sufferer was carefully tutored. I have a hard-headed, business friend who considers that doctors should be consulted only on such matters or occasions as marriages, births, and deaths. Or "matches, hatches, and dispatches," as he cheerfully describes them. Of equable temperament in most respects, he is attracted by the Maskelyne and Devant of the market-place when sometimes the attention of a qualified doctor would relieve him earlier. Especially in the North of England, where marriage is not always a leap in the dark, doctors might discuss previous illnesses and the future welfare and health of the patient more than is generally done at present. Put certain sequelæ in front of a fully qualified practitioner, and his opinion will bear the weight of criticism, and it is correct that as much consideration should be given to Polly and her past as judgment of her appearance and present condition. That some medical men do appreciate the necessity of studying the patient's view-point, however, is proved by the articles in the daily Press, which are calculated to fasten on to the imagination of the everyday type of person. Thus we find a physician discussing in simple language such diverse subjects as "Pneumonia and the V-neck Blouse," "Eye Transplantation," and "Psycho-analysis"—in its relation to the ringworm, or lugworm, or some poor worm.

Propaganda may be necessary and useful, but possibly etiquette prevents a specialist from modestly advertising his cleverness and disallows the services of a professional booster. But there are many doctors who, I feel sure, would be more popular and much more successful by becoming more and more approachable. They would then find the world in his shirt-sleeves awaiting them, anxious to get on with the next business.

H. L. Brunswick.

